# Numerical Cognition Based on Precise Counting with a Single Spiking Neuron

**DOI:** 10.1016/j.isci.2020.100852

**Published:** 2020-01-22

**Authors:** Hannes Rapp, Martin Paul Nawrot, Merav Stern

**Affiliations:** 1 Computational Systems Neuroscience, Institute of Zoology, University of Cologne, Zülpicher Straße 47b, 50923 Cologne, Germany; 2 Department of Applied Mathematics, University of Washington, Lewis Hall 201, Box 353925, Seattle, WA 98195-3925, USA

**Keywords:** Neuroscience, Cognitive Neuroscience, In Silico Biology

## Abstract

Insects are able to solve basic numerical cognition tasks. We show that estimation of numerosity can be realized and learned by a single spiking neuron with an appropriate synaptic plasticity rule. This model can be efficiently trained to detect arbitrary spatiotemporal spike patterns on a noisy and dynamic background with high precision and low variance. When put to test in a task that requires counting of visual concepts in a static image it required considerably less training epochs than a convolutional neural network to achieve equal performance. When mimicking a behavioral task in free-flying bees that requires numerical cognition, the model reaches a similar success rate in making correct decisions. We propose that using action potentials to represent basic numerical concepts with a single spiking neuron is beneficial for organisms with small brains and limited neuronal resources.

## Highlights

• A single spiking neuron can successfully learn to solve numerical cognition tasks
• The number of action potentials can represent numerosity
• Learning to count within few epochs allows generalization to unseen categories
• Counting with a single spiking neuron can solve numerical cognition tasks in insects

## Graphical Abstract

**Figure:**
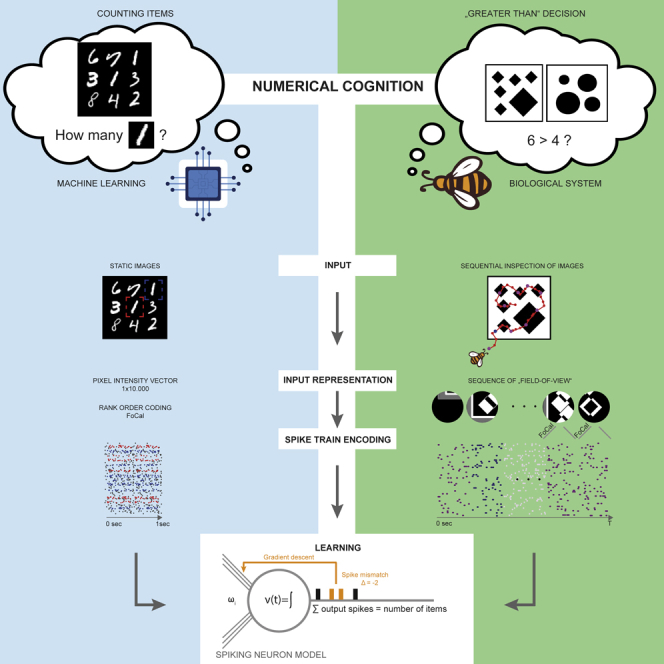

## Introduction

Insects have been shown to possess cognitive abilities (Chittka and Niven, 2009, Avarguès-Weber et al., 2011, Avarguès-Weber et al., 2012, Avarguès-Weber and Giurfa, 2013, Pahl et al., 2013). These include estimating numerosity (Rose, 2018, Skorupski et al., 2018), counting (Chittka and Geiger, 1995, Dacke and Srinivasan, 2008, Menzel et al., 2010), and other basic arithmetical concepts (Howard et al., 2018, Howard et al., 2019). How insects succeed in these cognitive tasks remains elusive. A recent model study by Vasas and Chittka (2019) suggested that a minimal neural circuit with only four rate-based neurons can implement the basic cognitive ability of counting visually presented items. The study implies that their minimal circuits can recognize concepts such as a “higher” or “lower” item number and “zero” (Howard et al., 2018) or “same” and “different” number of items (Avarguès-Weber et al., 2012) when combined with a sequential inspection strategy that mimics the behavioral strategy of insects during detection (Dacke and Srinivasan, 2008). The neural circuit studied in Vasas and Chittka (2019) was shown to successfully predict whether a particular feature (e.g. yellow) has been presented more or less often than a pre-defined threshold number, despite being presented in a sequence of other features and distractors. This circuit model was hand-tuned in order to successfully estimate numerosity in a numerical ordering task similar to Howard et al. (2018). This poses the question on how an efficient connectivity, which allows the network to estimate numerosity, could be learned by means of synaptic plasticity.

Numerosity estimation tasks that require counting the number of detected instances have also been researched in the field of computer vision, in particular in relation to object recognition tasks. Many resources have been devoted to train artificial neural networks to perform such tasks. Deep learning methods (Schmidhuber, 2015) in particular have been shown to be successful in object detection, and they enable counting by detecting multiple relevant objects within a static scene either explicitly (Ren et al., 2015) or implicitly (Lempitsky and Zisserman, 2010). However, these model classes are costly as they typically need to be trained on a very large number of training samples (in the millions) and often require cloud-computing clusters (Krizhevsky et al., 2012, Simonyan and Zisserman, 2014). Indeed, OpenAI, 2018 recently showed that the amount of computing power consumed by such artificial systems has been growing exponentially since 2012.

Clearly, insects with their limited neuronal resources cannot afford similar costly strategies and hence have to employ fundamentally different algorithms to achieve basic numerical cognition within a realistic number of learning trials. These biological algorithms might prove highly efficient and thus have the potential to inform the development of novel machine learning (ML) approaches.

A number of recent studies managed to train spiking neural networks with gradient-based learning methods. To overcome the discontinuity problem due to the discrete nature of action potentials some studies evaluated the post-synaptic currents in the receiving neurons for the training procedures (Nicola and Clopath, 2017, Huh and Sejnowski, 2017). Other studies used the timing of spikes as a continuous parameter (Bohte et al., 2000, O’Connor et al., 2017, Zenke and Ganguli, 2018), which led to synaptic learning rules that rely on the exact time interval between spikes emitted by the presynaptic and the postsynaptic neuron. These spike-timing-dependent plasticity (STDP) rules were found experimentally (Bi and mingPoo, 2001) and have gained much attention in experimental and theoretical neuroscience (Caporale and Dan, 2008, Song and Abbott, 2000). Other recent studies approached the problem by either approximating or relaxing the discontinuity problem (Zenke and Ganguli, 2018, Bengio et al., 2013) to enable learning with error backpropagation in spiking neural networks. Training single spiking neurons as classifiers has been proposed by Gütig and Sompolinsky (2006) and Memmesheimer et al. (2014). Closely related, Huerta et al. (2004) trained binary neurons to perform classification in olfactory systems.

Here, we study a biologically realistic spiking neuron model with a synaptic learning rule proposed by Gütig (2016). Our approach to numerical cognition takes advantage of the discrete nature of action potentials generated by a single spiking output neuron. The number of emitted spikes within a short time period represents a plausible biological mechanism for representing numbers. In a virtual experiment we train our neuron model to count the number of instances of digit 1 within a static image of multiple handwritten digits (LeCun and Cortes, 2010). The synaptic weights are learned from the observations, and thus our model overcomes the problem of hand tuning a single-purpose neuronal circuit. We then test the model on the same “greater than” task as in Vasas and Chittka (2019), but we use the model's ability of precise counting to derive the concept of “greater than.”

Because in the present work we are interested in estimating numerosity, the teaching signal in our model is a single integer value that is equal to the total number of relevant objects. To achieve successful training we introduce an improvement to the implementation in Gütig (2016) where the membrane potential was considered for gradient-based learning to overcome the spiking discontinuity problem. We show that our improved implementation to this approach allows to train the model with better generalization capabilities and also supports better the reliability of numerosity estimation under inputs with complex distributions, including noise distributions, as naturally present in the brain.

## Results

Our objective is the implementation of a spike-based method that can be trained to solve numerical cognition tasks. We employ the multispike tempotron (MST) (Gütig, 2016), a single leaky integrate-and-fire neuron model with a gradient-based local learning rule. We suggest a modified update rule of the learning algorithm that reduces the variance in training and test error. The model is subjected to three different tasks that progress from a generic spike-pattern detection problem to a biologically inspired dual choice task that mimics behavioral experiments in honeybees.

### Detection of SpatioTemporal Input Spike Patterns

We begin by considering the problem of detecting different events over time. A particular event is represented by a specific spatiotemporal spike pattern across a population of neurons that are presynaptic to the MST. These spike patterns are generic representations of events that could, for instance, represent sensory cues in an animal's environment.

We generated event-specific patterns of fixed duration (1 s) across 500 presynaptic input neurons using a gamma-type renewal process of fixed intensity (*λ* = 0.89 spikes per second) independently for each neuron (see Transparent Methods). The MST was presented with an input consisting of a sequence of different patterns on top of a noisy background that was simulated as independent gamma-type renewal processes of either constant or time-varying intensity (see Transparent Methods).

**Figure 1:**
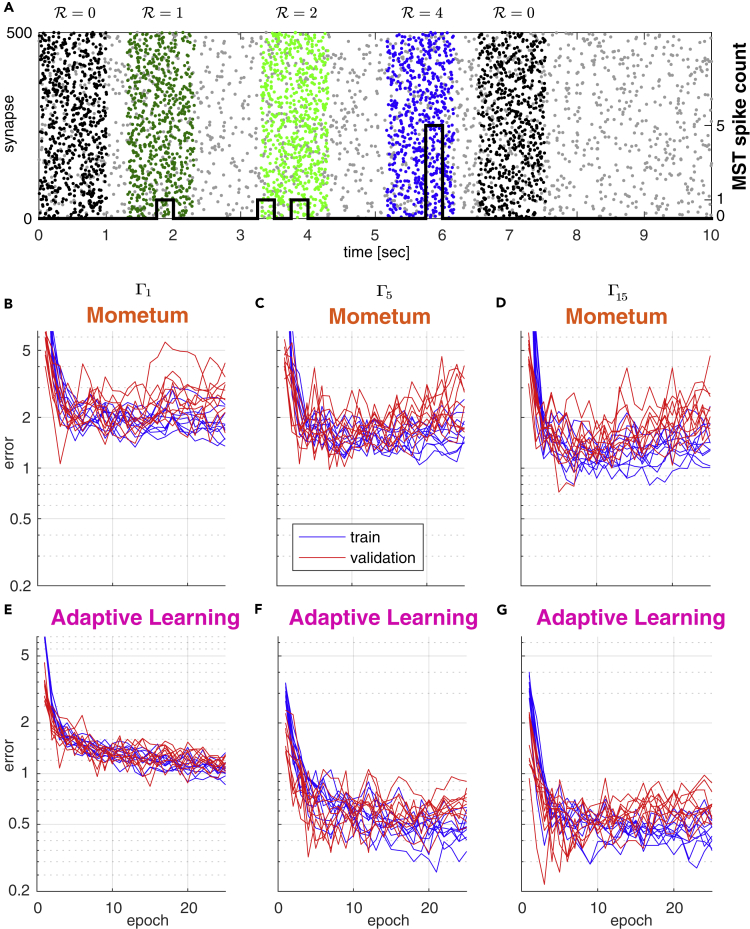
Comparison of Training Convergence for Momentum and Adaptive Learning under Different Background Noise Conditions (A) Sample input sequence: A 10-s-duration spike train input example. The spike train is composed of three patterns, each with a distinct target (dark green, light green, blue), background activity (gray), and two distracting patterns (black). Number of MST output spikes superimposed as black step function. The MST is supposed to fire ${\text{Σ}}_{i}R_{i}=7$ spikes over the whole sequence, R=0 spikes for distractors, and R=1,2 or 4 for the colored dark green, light green, and blue patterns accordingly. Patterns are simulated with gamma processes of different order (separate datasets): ${\text{Γ}}_{1}$ (Poisson), ${\text{Γ}}_{5}$, and ${\text{Γ}}_{15}$. Patterns are superimposed onto 10 s inhomogeneous Poisson background activity. (B–G) Training curves (blue) and validation curves (red) for 10 independent simulations of the (B and E) ${\text{Γ}}_{1}$ (Poisson), (C and F) ${\text{Γ}}_{5}$, and (D and G) ${\text{Γ}}_{15}$ patterns. (B–D) MST with Momentum-based learning implementation (Gütig, 2016). (E–G) MST with adaptive learning implementation. Learning (training) convergence shows larger variance when using Momentum as compared with using adaptive learning. The same is true for the validation (testing) error. This indicates that adaptive learning is capable of finding better optima compared with Momentum.

A single input trial is shown in Figure 1A. It accounts for the random occurrence of three different event-specific spatiotemporal spike patterns (in this specific example, each pattern occurring once) as indicated by different spike color and of distractor patterns occurring twice (black spikes). Gray spikes represent the background noise. Generally, for each trial of 10 *s* duration we randomly drew a number of pattern occurrences and pattern identities from a total of 9 possible patterns (five target patterns and four distractor patterns).

We first trained the original MST of Gütig (2016) to detect pattern occurrence. To each of the five event-specific patterns we assigned a specific target number of MST output spikes R (from 1 to 5). We did not assign a target to any of the distractor patterns (i.e. the MST was expected to produce zero output spikes in response to a distractor pattern). At the end of each training trial (one sequence of multiple patterns and distractors) the sum of actual output spikes was evaluated and compared with the desired number of output spikes determined by the trial-specific random realization of the input pattern sequence. The absolute difference between the desired and the actual spike count determined the training error in the range of $0-N\;\in N_{+}$. If the actual number of spikes was larger than the sum of the desired target spikes by some Δ_*k*_, a training step of the MST was performed toward decreasing its output spikes by the difference Δ_*k*_. Similarly, if the actual number was smaller than the sum of desired target spikes, a training step was performed to increase the MST's number of output spikes by Δ_*k*_. No training step was performed for correctly classified samples.

To analyze model performance we computed the training error and validation error for up to 25 training epochs (see Figures 1B–1D). Each training epoch consisted of a fixed, randomized set of 200 trials, and the validation set consisted of 50 trials. Both training error (blue) and validation error (red) dropped sharply with increasing number of training epochs and reached a plateau at about two spikes after ∼10 epochs, independent of the type of the gamma-order used for pattern generation (Figures 1B–1D).

### Local Synaptic Update Method Improves Performance and Robustness

Training and test errors exhibited a high variance across repeated models (Figures 1B–1D), indicating limited robustness of model performance. We therefore replaced the Momentum method for gradient descent implemented in the original work of Gütig (2016) by a synaptic specific adaptive update approach similar to RMSprop as proposed by Tieleman and Hinton (2012) (see Transparent Methods).

**Figure 2:**
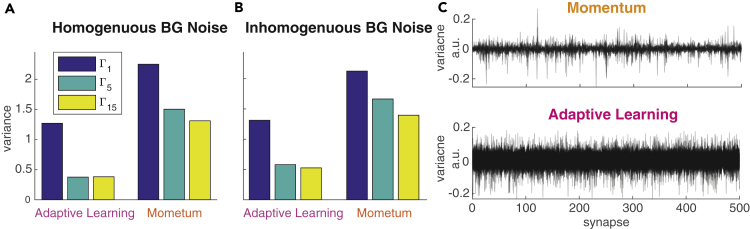
Training Convergence Properties of Momentum and Adaptive Learning (A and B) Variance of validation error measured at epoch 10 for datasets with homogeneous (A) and inhomogeneous (B) background noise. (C) Empirical analysis of the regularizing effect on the error variance. Weight changes Δ*ω*_*i*_ over all training steps (and all epochs) are collected for each synapse *ω*_*i*_. PCA is performed to reveal which synapses' weight changes show the largest variance over the training process. Large variance in Δ*ω*_*i*_ implies strong modification of a synapse. For both Momentum (top) and adaptive learning (bottom) the first 10 principal components are shown where *x* axes correspond to the synapses *ω*_*i*_ and *y* axis shows variance in total weight change per synapse *ω*_*i*_. The Momentum method tends to tune only a small subset of the available synapses strongly, whereas the adaptive learning method leads to modifications that are more uniformly distributed over all synapses and more broadly distributed in magnitude.

Although speed of convergence is similar when using the adaptive learning method compared with Momentum, we find that using adaptive learning results in less variant training error (Figures 1E–1G). This also holds for the variance of the test error on an independent validation set indicating better generalization capabilities to previously unseen inputs (Figures 1E–1G, 2A, and 2B). The adaptive, per synapse learning rate combined with exponential smoothing over past gradients has a regularizing effect and prevents the model from overfitting to the training data. We further conclude that the modified algorithm is potentially able to find better and wider optima of the error surface as compared with learning with Momentum. More importantly, this behavior is consistent and independent of the spike-generating process and noise level (Figures 2A and 2B).

At this point we cannot provide a theoretically grounded explanation for the regularizing effect we see when using adaptive learning instead of Momentum. Development of theoretically grounded explanations of the effects of different gradient-decent optimizers is a very recent and active research field in the Deep Learning community. To provide insights for the regularizing effect we therefore conducted an empirical analysis of the weight updates, as shown in Figure 2C. Specifically, we performed PCA on the weight changes Δ*ω*_*i*_ applied to all synapses over all training steps. The intuition here is that large variance in Δ*ω*_*i*_ implies strong modification of a synapse over the training process. Results of our analysis (Figure 2C) show that for the adaptive learning method the weight changes are more uniformly distributed over all synapses and more broadly distributed in magnitude. In contrast, with the Momentum method only a small subset of synapses is strongly modified. We conclude that distributing the updates uniformly over all synapses leads to a more deterministic convergence behavior toward good minima in the error surface, independently from the initial, random initialization of *ω*_*i*_. The results shown are obtained from a specific choice of meta-parameters (*α* = *γ* = 0.99, *λ* = 0.01), but we verified that it remains true over a broad range of possible values and combinations.

Moreover, we find that adaptive learning improves absolute performance converging to a smaller error independent of the actual gamma process when using the same values for the free meta-parameters for both methods. Although choosing different values for the meta-parameters results in different (and in some cases even lower) train and validation errors, our main result regarding the variance still holds. For subsequent tasks we used the MST with adaptive learning.

### Counting Handwritten Digits

**Figure 3:**
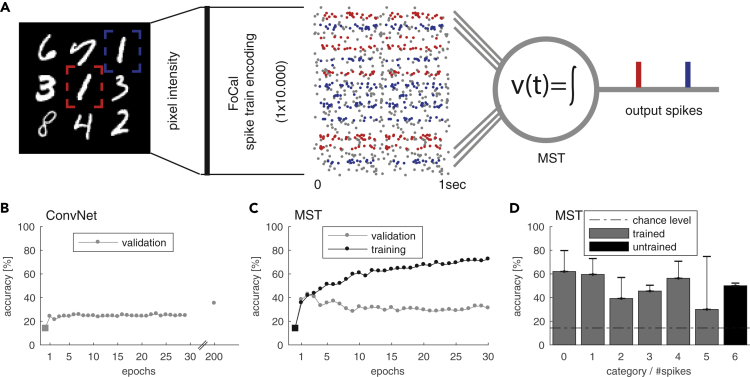
Counting of Visual Concepts with Spikes (A) Sketch of counting task. The goal of this task is to count the number of occurrences of digit 1 in an image of random MNIST digits. Example image (50×50 px) with multiple random digits from the counting MNIST dataset positioned within a 3×3 grid. The image is encoded into parallel spike trains by applying FoCal encoding, resembling a 4-layer early visual system with rank-order coding. The multivariate spike train converges onto the MST via 10.000 synapses. The MST is trained to elicit exactly *k* output spikes where *k* is equal to the number of digit 1 occurrences in the original image (here 2). (B) For reference we trained a ConvNet on the same raw images. Shown is the performance in terms of mean accuracy (five-fold cross-validation). After 200 training epochs the ConvNet reached ~40% accuracy. (C) Performance of the MST in terms of mean accuracy (five-fold cross-validation). The MST shows rapid learning reaching a similar level of accuracy as the ConvNet after 200 training epochs within only two to four training epochs. (D) Mean accuracy +std for the possible numbers of digit 1 present within a single image (categories). The MST is trained on samples of categories 0–5 to generate 0–5 output spikes respectively. The MST is then tested on the untrained category 6 and is able to generalize reasonably while the ConvNet, by design, cannot make predictions for this category.

We apply the MST model to the problem of counting the number of instances of digit 1 within an image showing several random handwritten MNIST digits (LeCun and Cortes, 2010). The digits are randomly positioned within a fixed 3×3 grid (Figure 3A). Each image can contain between zero and six instances of the digit 1 at one of the nine possible grid locations. To solve this problem with the MST we take the 50*x*50px input image and encode the entire image as a parallel spike train. To transform the image into a parallel spike train that can be fed into the MST model we use filter-overlap correction algorithm (FoCal) of Bhattacharya and Furber (2010). This method is an improved four-layer model of the early visual system using rank-order coding as originally proposed by Thorpe and Gautrais (1998). We then train the MST model to count the number of occurrences of digit 1 by generating one output spike for each instance of digit 1 (Figure 3A). We train the MST on targets 0–5 using five-fold cross-validation on 400 sample images. The learning rate is tuned manually to *λ* = 0.00002, which yields the best performance and training speed. For reference we compare the performance of the MST with a conventional computer vision model that uses a convolutional neural network (ConvNet) (Krizhevsky et al., 2012, Seguí et al., 2015, Fomoro, 2017, Kingma and Ba, 2014, Yu and Koltun, 2015). The ConvNet is trained similarly but provided 800 training samples and a larger learning rate of 0.01 to speed up the training process.

Counting, as a conceptual problem, is similar to a regression problem where we have no a-priori knowledge of the maximum number of desired targets present in an input. It is important to note that the ConvNet model used for comparison is built using prior knowledge about the distribution of the training set. The ConvNet is constrained to learn a categorical distribution over [0,5], where 5 is the maximum possible count of desired digits in the used training set of images. This has two implications. First, the ConvNet model will be unable to predict images that include more than five targets. However, in general for regression problems, the prediction targets are usually not bounded. Second, the counting error a ConvNet can make is constrained by the training bound, i.e. the maximum error is 5. In contrast, the MST model does not have any need for this prior knowledge or constraints. In principle it is capable of solving the general, true regression problem and can (after being trained) also make predictions for images that contain more than five occurrences of digit 1. It thus has to solve a more difficult learning problem. The maximum prediction error in this case is unbounded rendering the MST more vulnerable to prediction errors compared with the ConvNet. Figure 3B shows the performance of the ConvNet in terms of mean accuracy of correctly counted images. Despite the large learning rate, accuracy only slowly (but monotonically) improves over the course of 200 training epochs. In contrast, the performance of the MST in Figure 3C shows rapid learning, reaching similar mean accuracy as the ConvNet within only ∼3 training epochs. The MST reaches a performance above chance level for each of the trained target categories 0–5 (Figure 3D). It also performs above chance level for images that contain six targets. This indicates that the MST is not only learning a categorical distribution over 0–5, as is the case for the ConvNet but also generalizes to a larger, previously unseen number of targets. We want to emphasize that the MST performs better than the ConvNet despite the advantages given to the latter in the form of a larger number of training samples and a higher learning rate. The results are further summarized in Table 1.

**Table 1:** Results for MNIST Digit Counting MNIST Task

Counting MNIST Results: Counting Ones			
Model	#Parameters	RMSE (Mean ±std)	Accuracy (Mean ±std)
ConvNet (5 epochs)	11,079	1.70 ± 0.2083	23.00 ± 0.1746
ConvNet (100 epochs)	11,079	1.67 ± 0.3130	27.07 ± 0.5113
ConvNet (200 epochs)	11,079	1.02 ± 0.0768	40.97 ± 0.8136
**MST**_**adaptive**_**(~4 epochs)**	10,000	1.21 ± 0.2067	47.72 ± 3.2052

We evaluate each model in terms of root-mean-square error (RMSE) of the difference in actual and predicted number of digits (a lower RMSE indicates a better performance) and accuracy of correct digit count in images. Reported results are mean and standard deviation over a five-fold cross-validation.

During our experiments we found that the choice of the spike encoding method has a big impact on the MST's performance. It is possible that, by applying better or more efficient encoding algorithms, the performance of the MST model can be further improved.

### Insect-Inspired Numerical Cognition During Visual Inspection Flights

We now consider a biologically motivated task following Vasas and Chittka (2019) and the original experiment conducted in honeybees by Howard et al. (2018). The objective in this experiment is to perform a “greater than” dual choice task on two stimulus images that show varying numbers of geometric shapes (circles, squares, diamonds). The geometric shapes within a stimulus image are consistent, and the possible number of them range from 1 to 6.

**Figure 4:**
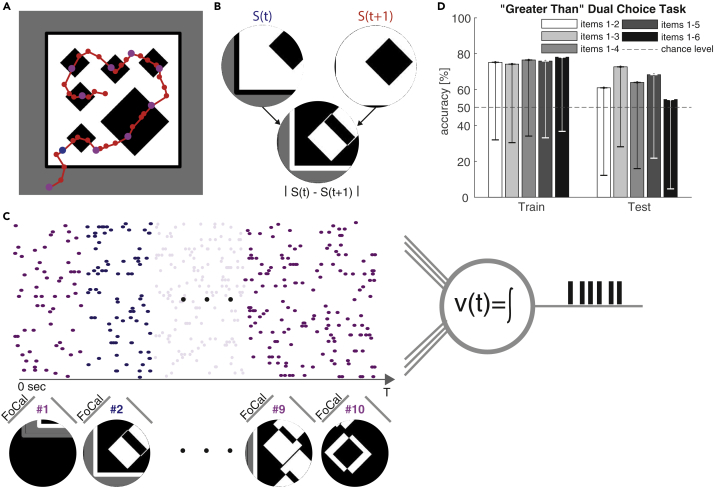
Dual Choice “Greater than” Task Performed on Geometric Shapes Using a Visual Inspection Strategy Observed in Honeybees (A) Sample stimulus image with six diamond shapes and inspection trajectory (red) of a honeybee. The trajectory is sampled at 40 points [Vasas and Chittka (2019)] (all dots on the trajectory) and sub-sample at 10 points for the MST (purple and blue dots). (B) Field of view (FOV) *S*(*t*) and *S*(*t*+1) of the honeybee during its inspection trajectory (at the blue dot and its subsequent red dot on the trajectory in (A), accordingly). Following the method of Vasas and Chittka (2019), input to the model is constructed as a derivative of the two subsequent FOV images: $FOV_{diff}=\left|S\left(t\right)-S\left(t+1\right)\right|$. (C) Sequences the *FOV*_*diff*_ are encoded into spatiotemporal spike patterns using rank-order coding (FoCal) and concatenated (without gaps) into the resulting parallel spike train. The MST is trained to match its number of output spikes to the number of geometric items in the original stimulus image shown in panel. (D)Performance in the “greater than” dual choice task. The MST output (number of spikes), ${\hat{y}}_{1},{\hat{y}}_{2}$ in response to two different stimulus images with number of items *y*_1_,*y*_2_, accordingly, is used and compared. When $\left(y_{1}<y_{2}\right)\wedge \left({\hat{y}}_{1}<{\hat{y}}_{2}\right)$ the decision is considered correct (and vice versa for $y_{1}>y_{2}$, for ${\hat{y}}_{1}={\hat{y}}_{2}$ a random decision was taken). Bars show mean accuracy −std and grouped by increasing maximum number of items present per image. Our results indicate that the MST can achieve mean accuracy that is comparable to that of honeybees reported in Howard et al. (2018).

In contrast to our previous task, here a stimulus image is not presented as single static input. Instead the input is a sequence of smaller images that mimic the 60^∘^ field-of-view (FOV) of honeybees hovering over the stimulus image at a distance of 2 cm (see Transparent Methods). The available corresponding dataset that consists of stimulus images and corresponding inspection flight trajectories recorded from behaving honeybees is highly imbalanced and limited to a total of 97 images. Figure 4A shows an example stimulus image with six diamond shapes and the inspection trajectory taken by one honeybee. This particular trajectory yields a sequence of ∼40 FOV images (red dots). Following the same procedure as Vasas and Chittka (2019), the absolute value of the derivative |S(t)−S(t+1)| of two subsequent FOV images S(t),S(t+1) is computed as input to the model (see Figure 4B). To reduce computational cost for our MST model and to unify the varying sequence length across all stimuli, we sub-sample the trajectories to length 10 (magenta dots). In Vasas and Chittka (2019) a rate-based model was used, and the FOV images were encoded into a univariate time-series (representing a rate) that is fed into the model as a single presynaptic input. Because our MST is a spiking model we have to encode each FOV image into a spike train. We apply the same encoding strategy as used before in the counting MNIST task: each FOV image is encoded as a parallel spike train of 10,000 synapses using FoCal. All encoded FOV images are combined into a long parallel spike train by concatenation (see Figure 4C).

The task is divided into two steps. The MST counts the number of geometric items present in a stimulus image. The resulting count numbers are then compared to solve the “greater than” dual choice task. This differs from the original behavioral task by Howard et al. (2018) in which the honeybees were directly trained on the “greater than” decision rather than on precise counting.

To this end we trained the MST (using 10-fold cross-validation) to match the number of generated output spikes to the number of geometric items present. To evaluate the dual choice task we took two random stimulus images with a different number of items *y*_1_,*y*_2_ and fed these images as input into the trained MST. We then compared the true item count with the predicted item count ${\hat{y}}_{1},{\hat{y}}_{2}$ of the MST. If $\left(y_{1}<y_{2}\right)\wedge \left({\hat{y}}_{1}<{\hat{y}}_{2}\right)$ it was considered a correct decision and vice versa when $y_{1}>y_{2}$. For undecidable cases where ${\hat{y}}_{1}={\hat{y}}_{2}$ a random decision was taken. This sampling process of decisions was repeated for 1,000 iterations. Our results (Figure 4D) show that the MST model is able to achieve comparable performance to the average performance of the honeybees (60%–70%) in the original task of Howard et al. (2018) in terms of mean accuracy of correct decisions. We want to emphasize that the MST performance could be achieved despite the very small and imbalanced training data. Moreover, the MST is trained on the problem of precise counting that is harder than the binary decision task. Although we have to acknowledge that the results show large error bars (due to the very limited training data), we conclude that our results provide a successful proof-of-concept. Using a larger and more balanced training set and better feature encoding would certainly reduce the variability and further improve the performance.

## Discussion

### Counting as a Basis for Numerical Cognition

Numerical cognition is a general term that covers several sub-problems, for example numerosity, counting, relations (greater/smaller than), basic arithmetical operations, and many more. Although each individual sub-problem might appear fairly trivial to us as humans, it is yet not clear how this could be realized computationally on the level of spiking neurons or networks thereof. Despite the simplicity of these sub-problems they do provide a foundation for more complex concepts that humans make heavy use of and are relevant for behavioral decision making. For example, if one is able to count entities it might only be a small step toward combining that information to perform more advanced concepts such as empirical statistics and estimating (discrete) probabilities. Although the specific symbolic math concepts are unavailable to animals, they are still able to show basic numerical cognition and evaluate basic probabilities (Howard et al., 2018, Howard et al., 2019, Avarguès-Weber et al., 2012).

A first objective of the present work was to study whether a single neuron model has the computational power to support numerical cognition tasks. Specifically, we addressed cue detection and counting by handling neuronal input such that it generates an output spike count that matches the number of relevant cues in its input. In order to achieve this computationally, the presynaptic weights of the neuron need to be tuned. Given the fact that the parameter space is very large and many possible solutions may exist, manually tuning the parameters is usually not possible. It is therefore desirable to implement a plasticity mechanism that allows the neuron to tune its weights by learning from examples.

In this work we have explored the MST developed by Gütig (2016). This spiking neuron model can be trained by gradient-descent to produce a precise number of output spikes in response to multiple occurrences of patterns embedded in the presynaptic input. Different patterns are assigned to different target numbers of output spikes per pattern occurrence. Gütig (2016) showed that the MST can learn to detect different spike input patterns. It can further assign the correct number of output spikes matching the targets of individual patterns. The MST learns this despite the fact that the teaching signal is only provided as a single scalar value that is equal to the sum over all targets presented sequentially in the input. Departing from the homogeneous Poisson process studied in Gütig (2016), we confirmed MST performance for biologically more realistic (Farkhooi et al., 2011, Mochizuki et al., 2016, Nawrot, 2010) gamma processes as generators for input patterns on non-homogeneous background.

### Adaptive Local Learning Rule Benefits Model Robustness

We introduced a modification to the update rule of synaptic weights Δ*ω*_*i*_. The *adaptive learning* introduces a dynamic, synapse-specific learning rate whose value at training step *t* depends on its history of values from previous training steps. We find that this modification allows the MST to learn a parameter set for the synaptic weights that shows less variability of the training and validation error as compared with the original *Momentum* method used in Gütig (2016). Low variability in validation error is generally a desired property for any learning algorithm because this commonly implies low variability in prediction or classification performance on new, unseen data.

At this point we are unable to provide a theoretically grounded explanation of the regularizing effect shown by *adaptive learning*. The deep learning community currently still lacks theoretical understanding of the effects of different gradient-descent optimizers, which is actively researched (Choromanska et al., 2015, Jin et al., 2017). We performed an empirical analysis of the weight changes Δ*ω*_*i*_ over the course of training. Specifically, we used PCA to analyze the variance of Δ*ω*_*i*_ for each synapse over all training steps. Our analysis reveals that for the *adaptive learning* a large number of weights are affected. In contrast, when using the originally proposed *Momentum* method, a much smaller subset of synapses show significant weight changes, and their distribution appears much more heavy-tailed with strong weight changes in few neurons. We conclude that modifying all synapses more uniformly appears to increase the likelihood that training converges toward good minima, independent from the initial random choice of *ω*_*i*_.

### Spike-Based Biological Learning versus Rate-Based Machine Learning

A second objective of this work was to explore possible advantages and disadvantages of a spike-based learning algorithm in comparison to a state-of-the-art deep learning architecture. Biological learning mechanisms enable animals to learn rapidly in a complex and dynamic environment. Instances where sensory cues coincide with reward or punishment during exploration may be sparse, i.e. they have to learn on very small sample sizes and slow learning could have fatal consequences. Single-trial learning, for instance, seems to be a fundamental ability found in different animals. Insects, for example, are able to form long-lasting associative memories upon a single coincident presentation of a sensory stimulus and a reinforcing stimulus (Pamir et al., 2014, Scheunemann et al., 2013, Zhao et al., 2019). Increased learning speed generally comes at the cost of increased generalization error and thus learning speed and high accuracy are in trade off.

We compared the biologically inspired spike-based learning algorithm of the MST with the deep learning architecture of a convolutional neural network, a standard computer vision model (ConvNet). We found that the MST is able to rapidly learn this task within ∼3 training epochs of 320 samples each to achieve a mean accuracy of ∼47% of correctly counted digits. Additional training did not improve accuracy. Conversely, the ConvNet, despite a 1000× larger learning rate and 100% more training samples per epoch, required >200 training epochs to achieve a similar accuracy. With additional training, the ConvNet achieved >80% accuracy for >5000 training epochs (not shown). Our results reflect a trade-off between very fast but less accurate learning with the spike-based MST method versus slow but increasingly accurate learning with the ConvNet. An additional aspect of biological relevance is the consumption of (computing) resources that are considerably higher for the ConvNet than for the single neuron MST. It is possible that in nature processing with spikes is generally more energy efficient, an important constraint in living organisms (Levy and Baxter, 1996, Niven and Laughlin, 2008, Niven, 2016).

Once trained, the ConvNet is only able to learn a categorical distribution over a fixed set of possible targets (here 0–5) that needs to be put into the design of the model a-priori. Similarly, previous related work of gradient-based learning in spiking network models is mostly concerned with solving classical classification tasks with pre-defined classes (Zenke and Ganguli, 2018, Bohte et al., 2000, Gütig and Sompolinsky, 2006, Memmesheimer et al., 2014). In this work we applied the single-neuron MST model to solve a regression problem. We show that the MST model does not have the limitation of the ConvNet. After being trained on targets 0–5 it was able to generalize to previously unseen images that contained digits 1 at 6 out of the 9 possible positions. This indicates that, in principle, the MST can solve full regression problems.

Differently from all other tasks presented in this work, the difficulty in this task is that each input stimulus is presented as a whole and not sequentially. This means that spike patterns associated with each occurring instance of digit 1 are distributed spatially (over different sets of synapses) instead of temporally. Due to the random positioning within the 3×3 grid, the patterns to be identified by the MST “*jump*” over different sets of synapses for different stimulus images that share the same training target make this task particularly hard to solve.

### Relational Operation Based on Counting

In the final task we went one step further and studied (precise) counting, as it allows other basic numerical cognition tasks to emerge. Assuming that a single neuron can count by relating the sum of its output spikes to the number of items present in a single stimulus, we show that this allows solving other numerical cognition tasks.

To this end we use a biologically motivated “greater than” dual choice task, performed by honeybees that employ a sequential inspection strategy. Honeybees are presented with stimulus images that show 1–6 different geometric shapes. Given two different stimulus images, the bees have to decide which of the two images contain more geometric items. Due to their limited FOV, the bees cannot perceive the stimulus image as a whole. Instead they perform a sequential inspection strategy, by hovering over the entire stimulus image. This results in a time series of FOV images, similar to a moving spot light. Using the MST, we approach this problem similarly and present a long parallel spike train that contains a sequence of FOV images.

In contrast to the honeybees the MST is trained to perform precise counting of the geometric shapes, similarly to the counting task we presented earlier. To perform the “greater than” dual choice task we present two different stimulus images and compare the number of output spikes of the MST. We show that the MST is able to achieve average success rates in terms of correct decisions that are comparable to those achieved by honeybees in the original experiment. Although our results do show much larger error bars than the honeybees, this is due to the following important difference that needs to be considered: the bees are explicitly trained on the (binary) “greater than” task. To the contrary, the underlying problem that the MST solves here is *precise counting*, which is harder to solve in general. Differently from Vasas and Chittka (2019), where input is provided as a univariate rate signal, our model uses parallel spike trains as input, which are derived from the FOV images. Although Vasas and Chittka (2019) used handcrafted features based on the assumption that the number of step-like changes in global image contrast is proportional to the number of scanned items, the MST has to learn which features are relevant and hence are useful to extract from the spatiotemporal input spike patterns. The MST has been trained on this task with a small dataset of ∼70 samples per trained model. Increasing the training data will very likely result in better and more robust performance as well as smaller error bars.

### Conclusion

Action potentials represent an elemental discrete quantity used for information processing in nervous systems. We conclude from our study that action potentials produced by a single spiking neuron can support basic arithmetic operation of counting. The MST is a powerful single-neuron method that can be trained to solve regression problems on multivariate synaptic input. We successfully applied the MST to perform basic numerical recognition tasks on complex and noisy input. We suggest that using spikes to represent numerosity with a single neuron can be a beneficial strategy especially for small-brained organisms, which economize on their number of neurons.

### Limitations of the Study

The MST learning rule used in this work requires differentiation of the membrane potential (see Transparent Methods), which is considered to be biologically implausible. Gütig (2016) suggested an approximate formulation of the learning rule that uses correlation-based learning of presynaptic spikes and postsynaptic voltage, which is considered biologically plausible. In order to ensure comparability of our results with the results in the original work we here used the gradient-based learning rule that was evaluated in Gütig (2016) for all the experiments presented. Although in this work we specifically focused on the computational capabilities of the single-neuron model, the same model and learning rule could also be used to construct more complex and layered networks as shown in Gütig (2016). We leave the study of multiple interconnected MST neurons for future research.

One weakness of the MST identified in the course of our study is a tendency for overfitting. This can, for instance, be inferred from the insect-inspired numerical cognition task, where the MST can be trained to reach >80% in accuracy of precise counting on the training set, but performance on the test set remains low or even drops below chance level (data not shown). This indicates that the MST tends to learn more about the samples of the training set compared with learning features that would generalize to the test set. This, to some extent, is also the case for the MNIST task. A potential solution to this problem could be to introduce explicit regularization terms in the MST learning rule, similar to approaches realized in deep learning algorithms. During our experiments we further found that the choice of method for the multivariate spike encoding of images has a big impact on learning and prediction performance. The particular encodings we have tried (using the same datasets) are encoding the intensity of each pixel independently by a 1-s-long spike train, generated by a renewal process with mean intensity equal to the pixel's intensity. This does not produce the ideal robust spike patters that can be learned by the MST. We predict that improved or more sophisticated spike-encoding methods will boost performances.

## Methods

All methods can be found in the accompanying Transparent Methods supplemental file.

## Data and Code Availability

To support further research we make our code and datasets publicly available at Rapp and Stern (2019).

## Supplemental Information

Document S1. Transparent Methods
