# Numerical Cognition Based on Precise Counting with a Single Spiking Neuron

**DOI:** 10.1016/j.isci.2020.101283

**Published:** 2020-06-27

**Authors:** Hannes Rapp, Martin Paul Nawrot, Merav Stern

## Main Text

(iScience *23*, 100852-1–100852-12; February 21, 2020)

In the originally published article, labels of Figures 1B-1D and 2A-2C contain spelling errors in important technical terms. Specifically, Figures 1B-1D, 2A and 2B state "Mometum" whereas the correct term is "Momentum," and the y-axis label of Figure 2C says "variacne" instead of "variance". Additionally, the title of Figures 2A and 2B have been corrected to proper spellings of "Homogeneous" and "Inhomogeneous." The corrections of these typos do not affect any of the reported results or conclusions of the paper but nevertheless should be stated correctly. The authors sincerely apologize to the readers for this unintended lack of thoroughness and any confusion that may have resulted from these typos.

**Figure:**
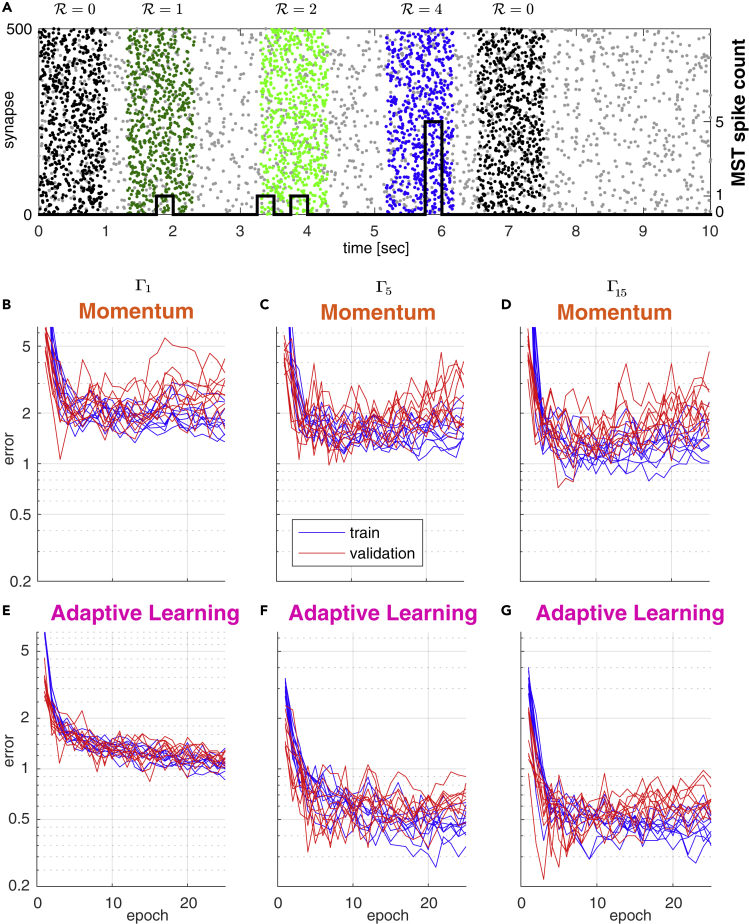
Figure 1. Comparison of Training Convergence for Momentum and Adaptive Learning under Different Background Noise Conditions (corrected)

**Figure:**
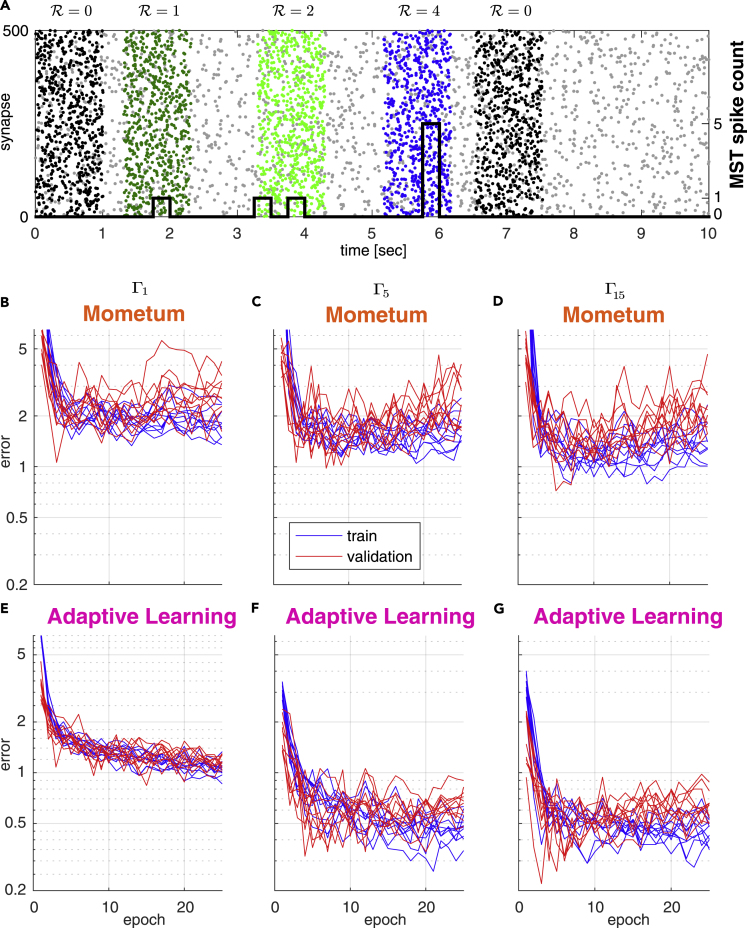
Figure 1. Comparison of Training Convergence for Momentum and Adaptive Learning under Different Background Noise Conditions (original)

**Figure:**
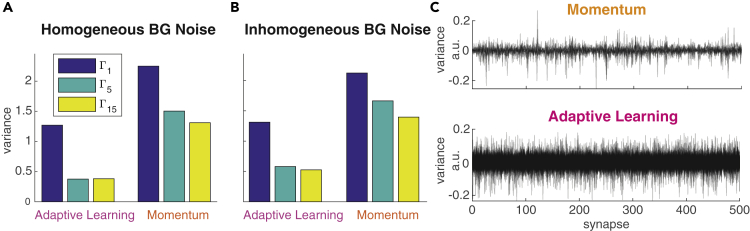
Figure 2. Training Convergence Properties of Momentum and Adaptive Learning (corrected)

**Figure:**
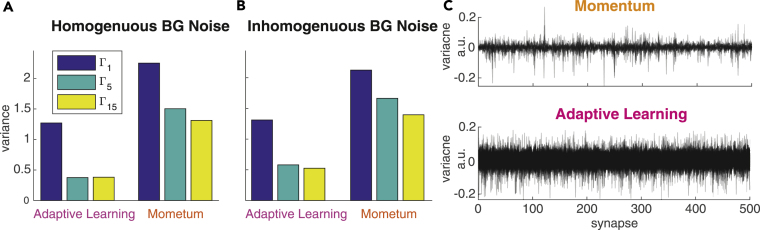
Figure 2. Training Convergence Properties of Momentum and Adaptive Learning (original)

