# Supplementary material for: Numerical Cognition Based on Precise Counting with a Single Spiking Neuron
Source: iScience. 2020 Jan 22;23(2):100852. doi: 10.1016/j.isci.2020.100852 (PMC7005464; doi:10.1016/j.isci.2020.100852)
Supplement: Document S1. Transparent Methods [file mmc1.pdf]

iScience, Volume *23*

Supplemental Information

Numerical Cognition Based on Precise Counting with a Single Spiking Neuron

Hannes Rapp, Martin Paul Nawrot, and Merav Stern

Supplemental Information

Transparent Methods

To support further research we make our code and data-sets publicly available at Rapp and Stern (2019).

Multispike Tempotron Model

The Multi-Spike Tempotron (MST) is a current-based leaky integrate-and-fire neuron model (Gütig, 2016). Its membrane potential, $V(t)$, follows the dynamical equation:

$$V(t) = \underbrace{V_{rest}}_{:=0} + \sum_{i=1}^{N} \omega_i \sum_{t_i^j < t} \overbrace{K(t - t_i^j)}^{\text{exp. PSP kernel}} - (\underbrace{\vartheta}_{:=1} - V_{rest}) \sum_{t_s^j < t} e^{-\frac{t - t_s^j}{\tau_m}} \tag{1}$$

where t_i^j denotes the time of spike number j from the input source (presynaptic) number i, and t_s^j denotes the time of postsynaptic spike number j of the Tempotron neuron model. For mathematical convenience the resting potential is chosen to be $V_{rest} = 0$ and the spiking threshold $\vartheta = 1$. Thus equation 1 can be simplified to:

$$V(t) = \overbrace{\sum_{i=1}^{N} \omega_i \sum_{t_i^j < t} K(t - t_i^j)}^{\text{unreset sub-threshold potential } V_0} - \vartheta \sum_{t_s^j < t} e^{-\frac{t - t_s^j}{\tau_m}} \tag{2}$$

Every input spike at t_i^j contributes to the postsynaptic potential (PSP) by the following causal kernel:

$$K(t - t_i^j) = \begin{cases} V_{norm}(e^{-\frac{t - t_i^j}{\tau_m}} - e^{-\frac{t - t_i^j}{\tau_s}}) & \text{if } t \geq t_i^j \\ 0 & \text{if } t < t_i^j \end{cases} \tag{3}$$

multiplied with the synaptic weight ω_i of input synapse i. These synaptic input weights are learned via the gradient decent algorithm. The kernel is normalized to have its peak value at 1 with $V_{norm} = \frac{\eta^{\frac{\eta}{(\eta-1)}}}{(\eta-1)}$ and $\eta = \frac{\tau_m}{\tau_s}$ where τ_m and τ_s are the membrane time constant and the synaptic decay time constant. The kernel is made causal by setting it to 0 for $t < t_i^j$. When $V(t)$ crosses the spiking

threshold ϑ the neuron emits a spike and is reset to $V_{rest} = 0$ by the last term in equation 2.

In order to have the neuron emit the required number of k postsynaptic spikes in response to some presynaptic spike pattern the weights ω_i are modified. Since the required number of postsynaptic spikes are non-differentiable discrete numbers the gradient for adjusting the weights is derived from the spiking threshold using an auxiliary objective function, the spike-threshold surface (STS). The STS is a step function $\mathbb{R}^+ \mapsto \mathbb{N}_0$, which maps each threshold value ϑ to the number of output spikes ($\vartheta \mapsto STS(\vartheta)$) that will be generated by the neuron with this threshold value. The STS for a presynaptic input can be described by the decreasing sequence of critical thresholds values ϑ_k^*:

$$\vartheta_k^* = sup\{\vartheta \in \mathbb{R}^+ STS(\vartheta) = k\}, k \in \mathbb{N} \tag{4}$$

The critical threshold ϑ_k^* denotes the threshold value at which the neuron's number of generated output spikes jumps from $k-1$ to k. The number of generated output spikes remains constant when ϑ is between two critical threshold values: $STS(\vartheta_{k+1}^* < \vartheta < \vartheta_k^*) = k$. Additionally, a neuron does not fire any output spike if its threshold is larger than the maximum postsynaptic voltage (V_{max}). In this case the STS is zero: $STS(\vartheta > V_{max}) = 0$. The first output spike is generated when $\vartheta = V_{max}$, thus the critical threshold for $k = 1$ spike is $\vartheta_1^* = V_{max}$. Generally, all ϑ_k^* are voltage values and can be described by the neuron's membrane equation 2 which is a function of the synaptic weights ω_i of the neuron. Hence, all critical thresholds are also a function of ω_i and thus differentiable with respect to them. The goal is to adjust ϑ_k^* (by modifying the synaptic weights ω_i) whenever the number of generated spikes does not match the desired training target. In our case, the specific k of desired output spikes is provided as supervised teaching signal. For each presynaptic input where the number of output spikes did not match the desired training target a training step is performed to adjust the number of output spikes towards k: $\Delta k = |k_{generated} - k_{target}|$ and $\eta = sign(k_{generated} - k_{target})$ indicates whether the neuron should increase or decrease its number of output spikes by Δk.

To simplify notation, from now on we denote ϑ^* as the desired critical threshold, e.g. $\vartheta^* = \vartheta_k^*$ for the desired k of a specific presynaptic input.

The gradient of the critical threshold can be found by:

$$\Delta\omega = \eta\lambda\vec{\nabla}_{\vec{\omega}}\vartheta^* \tag{5}$$

Where $\eta \in \{-1, 1\}$ controls whether to increase or decrease the number of output spikes towards the k required spikes, λ is the learning rate parameter that controls the size of the gradient step to take in each training step and $\vec{\nabla}_{\vec{\omega}}\vartheta^*$ is the gradient of the critical spiking threshold with respect to the synaptic weights. To evaluate the expression in eq. 5 the properties of the critical spike time t^* is used where by definition of the neuron equation 2 and ϑ^* the following identity is satisfied:

$$\vartheta^* = V(t^*) = V(t_s^j) \quad \text{where } t_s^j \text{ are all spike times before } t^* \tag{6}$$

In what follows a recursive expression is derived for the gradient in equation 5 using equations 2 and 3. For notional clarity the recursive expression for the gradient is derived for a single component ω_i of the vector $\vec{\omega}$. The generalization to $\vec{\omega}$ is immediate.

Let m denote the the number of output spikes the neuron fires before t^*: $t_s^j < t^*$ for $j \in \{1, ..., m\}$. Using the identities in 6, for each synapse i the derivative of ϑ^* has the following properties:

$$\vartheta_i^{*\prime} \equiv \frac{\mathrm{d}}{\mathrm{d}\omega_i}\vartheta^* = \frac{\mathrm{d}}{\mathrm{d}\omega_i}V(t^*) = \frac{\mathrm{d}}{\mathrm{d}\omega_i}V(t_s^j) \tag{7}$$

And the derivative of ϑ^* follows the equation:

$$\vartheta_i^{*\prime} = \frac{\partial}{\partial\omega_i}V(t^*) + \sum_{j=1}^{m}\frac{\partial}{\partial t_s^j}V(t^*)\frac{\mathrm{d}}{\mathrm{d}\omega_i}t_s^j \tag{8}$$

In the last equation the vanishing term $\frac{\partial}{\partial t^*}V(t^*)\frac{\mathrm{d}}{\mathrm{d}\omega_i}t^* = 0$ has been dropped. This relationship is true because $V(t^*)$ is either a local maximum with $\frac{\partial}{\partial t^*}V(t^*) = 0$ or t^* is the arrival time of an inhibitory input spike that does not depend on ω_i.

Similarly for each $k \in \{1,..,m\}$ the following relationship holds:

$$\frac{\mathrm{d}}{\mathrm{d}\omega_i}V(t_s^k) = \frac{\partial}{\partial\omega_i}V(t_s^k) + \sum_{j=1}^{k}\frac{\partial}{\partial t_s^j}V(t_s^k)\frac{\mathrm{d}}{\mathrm{d}\omega_i}t_s^j \tag{9}$$

from which the following equations are obtained:

$$\frac{\mathrm{d}}{\mathrm{d}\omega_i}t_s^k = \frac{1}{\dot{V}(t_s^k)}\left[\vartheta_i^{*\prime} - \frac{\partial}{\partial\omega_i}V(t_s^k) - \sum_{j=1}^{k-1}\frac{\partial}{\partial t_s^j}V(t_s^k)\frac{\mathrm{d}}{\mathrm{d}\omega_i}t_s^j\right] \tag{10}$$

$$\dot{V}(t_s^k) = \frac{\partial}{\partial t}V(t)\bigg|_{t=t_s^{k-}} \text{evaluated from the left before spike reset} \tag{11}$$

To solve equation 8 for $\vartheta_i^{*\prime}$, the right hand side of eq 10 is refactored to:

$$\frac{\mathrm{d}}{\mathrm{d}\omega_i}t_s^k = \frac{1}{\dot{V}(t_s^k)}\left[\vartheta_i^{*\prime}A_k + B_k\right] \tag{12}$$

The coefficients A_k, B_k are given by the following recursive equations:

$$A_k = 1 - \sum_{j=1}^{k-1}\frac{A_j}{\dot{V}(t_s^j)}\frac{\partial}{\partial t_s^j}V(t_s^k) \tag{13}$$

$$B_k = -\frac{\partial}{\partial\omega_i}V(t_s^k) - \sum_{j=1}^{k-1}\frac{B_j}{\dot{V}(t_s^j)}\frac{\partial}{\partial t_s^j}V(t_s^k) \tag{14}$$

Similarly for t^* the analogous recursion formula is defined:

$$A_* = 1 - \sum_{j=1}^{m}\frac{A_j}{\dot{V}(t_s^j)}\frac{\partial}{\partial t_s^j}V(t^*) \tag{15}$$

$$B_* = -\frac{\partial}{\partial\omega_i}V(t^*) - \sum_{j=1}^{m}\frac{B_j}{\dot{V}(t_s^j)}\frac{\partial}{\partial t_s^j}V(t^*) \tag{16}$$

Inserting equation 12 into 8 the derivative of $\vartheta_i^{*\prime}$ for each vector component i of ω can be expressed as:

$$\vartheta_i^{*\prime} = -\frac{B_*}{A_*} \tag{17}$$

To calculate A_* and B_* all times $t_x \in \{t_s^1, t_s^2...t_s^m, t^*\}$ are considered at which the voltage reaches the spiking threshold ϑ. At these time points, due to the spiking and reset, the membrane potential equation 2 reduces to the form:

$$V(t_x) = \frac{V_0(t_x)}{C_{t_x}} \tag{18}$$

with

$$V_0(t) = \sum_{i=1}^{N} \omega_i \sum_{t_i^j < t} K(t - t_i^j) \quad \text{unreset sub-threshold potential} \tag{19}$$

$$C_{t_x} = 1 + \sum_{t_s^j < t_x} e^{-\frac{t_x - t_s^j}{\tau_m}} \tag{20}$$

and gives the following derivatives:

$$\frac{\partial}{\partial \omega_i} V(t_x) = \frac{1}{C_{t_x}} \frac{\partial}{\partial \omega_i} V_0(t_x) \tag{21}$$

$$= \frac{1}{C_{t_x}} \sum_{t_i^j < t_x} K(t_x - t_i^j) \tag{22}$$

$$\frac{\partial}{\partial t_s^k} V(t_x) = \frac{-V_0(t_x)}{C_{t_x}^2} \frac{e^{-\frac{t_x - t_s^k}{\tau_m}}}{\tau_m} \quad \text{for } t_s^k < t_x \tag{23}$$

$$\dot{V}(t_x) = \frac{1}{C_{t_x}^2} \left[C_{t_x} \frac{\partial}{\partial t_x} V_0(t_x) + \frac{V_0(t_x)}{\tau_m} \sum_{t_s^j < t_x} e^{-\frac{t_x - t_s^j}{\tau_m}} \right] \tag{24}$$

Where in our implementation the temporal derivative $\dot{V}(t_x)$ is estimated numerically instead of using its analytical expression.

Momentum and Adaptive learning

The learning rate λ is global for all synaptic weights. Hence, the gradient descent takes an equal size step along all directions. If this parameter is too small the training process will take very long, but if it's too big the algorithm might miss an optimum within the error surface and never converge to a good solution. Hence, tuning this learning rate is important to achieve decent training speed. A possible approach (Gütig, 2016) to avoid these problems is to update the weights according to exponential moving average of current and past gradients (up to training step t), using the *Momentum* heuristic:

$$\begin{aligned} \Delta\omega^{Momentum} &= \alpha \Delta\omega(t-1) + \Delta\omega(t) \\ &= \alpha \Delta\omega(t-1) + \eta\lambda \vec{\nabla}_{\vec{\omega}} \vartheta^*, \end{aligned} \tag{25}$$

where α is the *Momentum* meta-parameter to control the exponential smoothing effect. In practice, a common heuristic in the machine learning community is to choose α's value as 0.999 while tuning the global learning rate λ.

Adaptive input weight learning and gradient smoothing

We propose here to use an adaptive learning approach for the weight updates instead of the *Momentum* heuristic. The proposed algorithm fits each input synapse with its own update rate and by doing so it takes into account that each synapse contributes to the overall update with a different level of importance. For example, updates should be larger for directions that provide more consistent information across examples. The RMSprop (Root Mean Square (back-)propagation) (Tieleman and Hinton, 2012) is a possible approach to achieve this. It was successfully used in deep learning for training mini-batches. It computes an adaptive learning rate per synapse weight ω_i as a function of its previous gradient steps :

$$\begin{aligned} v_i(t) &= \gamma v_i(t-1) + (1-\gamma)(\Delta\omega_i(t))^2 \\ \Delta\omega_i^{Adaptive}(t) &= \frac{\eta\lambda}{\sqrt{v_i(t)}} \vec{\nabla}_{\omega_i} \vartheta^* \end{aligned} \tag{26}$$

The dynamical variable $v_i(t)$ gives the synapse specific (e.g. local) learning rate for the current training step t. The value of this variable depends on the exponential moving average of current and past squared gradients (up to training step t). The meta-parameter γ controls the degree of exponential smoothing similarly to α of the Momentum method above. Setting $\gamma = 1$ would be similar to vanilla gradient descent where only the gradient of current training step t is used to update. In practice a common heuristic for the choice of γ in the deep learning community (also suggested by Tieleman and Hinton (2012)) is 0.999 and instead only tuning the global learning rate λ.

At this point we cannot provide a theoretically grounded explanation for the regularizing effect we see and report in the results section when using adaptive learning instead of Momentum. Theoretically grounded explanations of the effects of different gradient-decent optimizers are a very recent and ongoing research field in the machine learning community. We thus conducted an empirical analysis of the weight updates and report our findings in Figure 2C and conclusions in the discussion.

Detection of spatio-temporal input spike patterns.

In this task we study the general case of counting arbitrary, task dependent patterns. To this end we use 1sec long spike trains generated from point processes as a model of complex spatio-temporal patterns that represent features of task dependent activity. An input to the MST model consists of a sequence of such patterns, each of which assigned with a specific target $\mathcal{R}_i$. The patterns are superimposed onto a 10sec long spike train of background activity. Similar to the task in (Gütig, 2016) the MST model is trained to respond with spikes for each pattern occurrence where the number of spikes per pattern depends on its assigned target $\mathcal{R}_i$. For each data-set a training set of 200 samples and a separate validation set of 50 samples is generated. Each pattern is associated with a fixed, positive integer target $\mathcal{R}_i \in [0, 9]$. For each data-set the patterns are generated from a different renewal process. Out of the 9 patterns, 5 patterns are considered to be *task-related* and are associated with some positive target $\mathcal{R}_i$. The remaining 4 patterns are considered to be distractor patterns with target 0. The training target for each of such input spike train is determined as the sum over all individual targets $\Sigma_i \mathcal{R}_i$ of each occurring pattern.

For each data-set, at the end of each training epoch, the error in the MST performance is calculated as the mean absolute difference between the target input spike count and the actual MST response across all training trails (training error) or testing trials (validation error),

It has been shown that in-vivo cortical spiking activity is typically more regular than Poisson (Mochizuki et al., 2016; Nawrot, 2010). In general any correlated stimuli input is expected to deviate from Poisson (Farkhooi et al., 2011). Moreover, input is generally non-homogenous, i.e. time-varying. However in (Gütig, 2016) only homogeneous Poisson statistic of input patterns and background were considered.

All patterns are generated as 1sec long spike trains by drawing instantaneous firing rates from three different point processes (renewal processes): Γ_1 representing the homogeneous Poisson process, Γ_5, and Γ_{15} represent Gamma-Processes with a fixed intensity (or rate) of $\lambda = 0.89$ spike events per second.

Input spike trains of 10 sec duration and 500 presynaptic inputs are generated by simulating a 10 sec long spike train of background activity using renewal processes and patterns are superimposed onto this background activity. The number of patterns to appear within a sequence is drawn from a Poisson distribution of mean 5 patterns. These patterns are randomly positioned in time within those 10 sec but are not allowed to overlap (an example of an input spike-train is shown in fig. 1A).

We evaluate learning under different noise conditions, where one condition uses homogenous Poisson background activity and the other condition uses inhomogenous Poisson background activity. The homogenous background activity is drawn from a stationary Poisson process ($\lambda = 0.3$ spikes/sec) while for the inhomogenous case the instantaneous firing rates are slowly modulated by $\lambda(t) = sin(\frac{10\pi}{10000}t) + (\frac{4\pi}{10}\xi(t))$ where $\xi(t)$ is noise drawn from a standard normal distribution.

The free meta-parameters for Momentum and adaptive learning are set to be $\alpha = 0.999$ and $\gamma = 0.999$ respectively. These are heuristic values taken from current deep learning frameworks and in practice are treated as constant parameters. Thus, the only real free parameter is the global learning rate λ. Since the objective of this task is to study the effect of the two different update methods, we are not concerned to determine the optimal learning rate that would give the best possible, absolute numbers in terms of training error. The described effect in the results section is independent of the specific choice we made $\lambda = 0.001$, although the absolute numbers vary.

Counting handwritten digits

This task considers the problem of estimating numerosity. Specifically the problem of counting the occurrences of digit 1 within an image showing 9 random MNIST (LeCun and Cortes, 2010) digits positioned within a $3x3$ grid. Following Seguí et al. (2015) we generated new images of size 50x50 pixels. Each image is subdivided into a $3x3$ grid where each grid cell shows a randomly chosen (with replacement), single MNIST digit. Out of the 9 possible cells, up to 6

cells can be occupied by digit 1. This yields samples with possible targets from $0 - 6$. The generated data set is only roughly balanced, containing ~ 200 samples for each target $0 - 6$. The model is supposed to learn to count the number of occurrences of the digit 1 by generating one output spike per each occurrence. The training target is provided by a single scalar label of the number of digits 1 in the image. All models are trained using 5-fold cross-validation. While the training set for the MST model comprises 400 samples, the ConvNet is provided with 800 samples. Additionally, the ConvNet is provided with a much larger learning rate of $\lambda = 0.01$ to accelerate training, while the MST is manually tuned to use learning rate of $\lambda = 0.00002$. To train the ConvNet we use the ADAM (Kingma and Ba, 2014) optimizer which has been found to be an effective optimizer for training ConvNets. For the MST model we use our adaptive learning rate method where the meta-parameter is set to $\gamma = 0.999$. The MST model is trained for max. 30 epochs as it does not improve further after this. The ConvNet is trained for max. 200 epochs. For all models, the training is considered to be converged at that epoch before the validation error diverges for the first time. While the ConvNet shows monotonic decrease of validation error, the MST fluctuates.

For the Multi-Spike Tempotron the images have to be encoded as spike trains. This is done by using *Filter-Overlap Correction Algorithm* (FoCal) (Bhattacharya and Furber, 2010), a 4-layer model of the early visual system that uses an improved rank-order coding originally proposed by Thorpe and Gautrais (1998). Encoding a single $50x50$px image thus yields a spike train with $4 \times 50^2 = 10000$ synapses. The encoding algorithm makes use of spatial correlations in order to reduce the amount of redundant information. This is similar to the convolutional filters embedded in current deep neural networks (Simonyan and Zisserman, 2014; Krizhevsky et al., 2012). For reference, we train a conventional ConvNet architecture that has been shown to successfully accomplish this task when trained on 100000 samples. The architecture uses several layers (conv1 - MaxPool - conv2 - conv3 - conv4 - fc - softmax) and includes recently discovered advances like strided and dilated convolutions (Yu

and Koltun, 2015).

The free meta-parameters for the MST model, Adaptive learning parameter γ and global learning rate λ, are set to be $\gamma = 0.999$ and $\lambda = 0.0001$. This learning rate has been determined manually, by step-wise decreasing from 0.1 by factor of 10 until reaching the best trade-off between learning speed and convergence of validation error. For the choice of γ we refer to the explanation given in the method section above.

Insect-inspired numerical cognition during visual inspection flights

Following Vasas and Chittka (2019); Howard et al. (2018) we consider estimation of numerosity of geometric shapes during a sequential inspection strategy employed by insects. We use 97 sample trajectories from sequential inspection flights from real honeybees, taken from supplements of Vasas and Chittka (2019). The available trajectories cover samples from 0 to 6 items (we removed 0 since it only had a single trajectory). Following Vasas and Chittka (2019) the trajectories have been used to extract a sequence of single images with a field of view (FOV) of 60° and 2cm distance to the inspected image. Thus each time point of a scanning trajectory yields a $183x183$ pixel image. Particularly, the absolute difference of each image S between two successive time points t and $t+1$ (1st derivative) of the trajectory is used: $FOV_{diff} = |S(t) - S(t+1)|$. While the proper way would be to use $|S(t-1) - S(t)|$ we decided to exactly follow the method used in Vasas and Chittka (2019). Differently from Vasas and Chittka (2019) the sequence of derivative images is down sampled to obtain sequences of equal length of 10 derivative images. This is done to reduce the computational cost as well as removing some redundant information from overlapping field of views between two successive time steps (a very coarse approximation of a working memory). All images are further down-scaled by factor 0.25 to $46x46$ pixels. This additional preprocessing is done to further reduce computational cost and to reduce the number of free parameters (synapses) in the MST model. To obtain spike trains from the image sequences, each FOV_{diff} image is encoded as a short parallel spike train using Filter-overlap Correction (FoCal) algorithm

(Bhattacharya and Furber, 2010). FoCal resembles a 4-layer early visual system and is an improved rank-order coding scheme of images originally proposed by Thorpe and Gautrais (1998). The resulting parallel spike trains per FOV_{diff} image are finally concatenated (without gaps) into a single long parallel spike train. Using this encoding results in parallel spike trains with 8468 input synapses to the MST. The MST model is trained (supervised) to fit its numbers of output spikes to the precise item count of geometric shapes. We used the adaptive learning method described above with $\gamma = 0.999$ (see explanation in methods section above), $\lambda = 0.00002$ (manually tuned) and performed a 10-fold, stratified cross-validation and trained for max. 25 epochs. We consider a model's training to be converged at that epoch before the validation error diverges for the first time. This generally was the case after 4-7 epochs. We assess the performance on a 'greater than' dual choice task following the original experiments of Howard et al. (2018). To this end, we randomly choose two independent samples of numerosity (y_1, y_2) and feed the corresponding images into a randomly chosen, trained instance of the MST (10-fold cross-validation yields 10 independent models in total). A prediction by the MST $\hat{y}_1, \hat{y}_2$ is considered to be correct if $(y_1 > y_2) \wedge (\hat{y}_1 > \hat{y}_2)$ (and vice versa). In undecidable cases where $\hat{y}_1 = \hat{y}_2$ a random decision is made (coin-flip). This sampling process is repeated for 1000 random pairs, independently and separately for the training and testing data sets.
